# Chronic Cardiac Resynchronization Therapy-Defibrillator Infection Leading to Sepsis and Bilateral Septic Pulmonary Emboli: A Case Report

**DOI:** 10.7759/cureus.86142

**Published:** 2025-06-16

**Authors:** Tinatin Makaridze, Tinatin Jomidava, Nikoloz Kvaratskhelia, Khatuna Jgushia, Ana Abesadze, Roin Rekvava

**Affiliations:** 1 Internal Medicine, American Hospital Tbilisi, Tbilisi, GEO; 2 Pharmacology, European University, Tbilisi, GEO; 3 Microbiology, Tbilisi State Medical University, Tbilisi, GEO; 4 Infectious Disease, American Hospital Tbilisi, Tbilisi, GEO; 5 Urology, Caucasus Medical Center, Tbilisi, GEO; 6 Anatomy, Georgian National University, Tbilisi, GEO; 7 Clinical Skills, Georgian National University, Tbilisi, GEO; 8 Electrophysiology, American Hospital Tbilisi, Tbilisi, GEO

**Keywords:** cardiac device-associated endocarditis, cardiac implantable devices, cardiac resynchronization therapy (crt), septic emboli, septic pneumonia, septic pulmonary embolism

## Abstract

Cardiac implantable electronic devices (CIEDs), including cardiac resynchronization therapy-defibrillators (CRT-Ds) and other pacemaker systems, are widely used in patients with varying degrees of heart failure to improve cardiac function and reduce the risk of sudden cardiac death. Despite their life-saving benefits, infections related to CRT-Ds present significant clinical challenges. These infections can lead to serious complications, including endocarditis, pocket infections, and device-associated bloodstream infections, often resulting in prolonged hospitalization and potentially life-threatening outcomes. Key risk factors include comorbidities such as diabetes, use of immunosuppressive therapy, and procedural complications. Early recognition and timely intervention, typically involving antibiotic therapy and/or device removal, are essential to improving patient outcomes. In this report, we describe a rare case of chronic CRT-D infection complicated by bilateral septic pulmonary emboli and sepsis, which was successfully managed through a multidisciplinary approach involving device extraction and targeted antibiotic therapy with vancomycin and cefepime.

## Introduction

Cardiac implantable electronic devices (CIEDs) play a critical role in managing various cardiac conditions, including arrhythmias, heart failure, and other cardiac abnormalities. Their use has expanded substantially in recent years, paralleling the increasing number of device implantations [1]. CIEDs have significantly improved survival rates and quality of life for many patients; however, complications remain a significant clinical concern, with infection being among the most serious. Device-related infections can result in severe and potentially life-threatening outcomes [2]. Infection rates following CIED implantation have been reported to range from 5% to 20% [3], a particularly alarming statistic given that infection incidence is rising faster than implantation rates. This trend suggests that factors beyond procedural frequency, such as patient comorbidities, aging populations, healthcare-associated risks, and increasing device complexity, may contribute to heightened susceptibility. Patients receiving more complex systems, such as cardiac resynchronization therapy (CRT) devices, appear especially vulnerable. Reported infection rates are 1.6% over a 2-year follow-up and 1.9% over 3.4 years for CRT with pacemakers (CRT-Ps), compared to 3.1% over a 3.4-year follow-up and 8.6% over 2.6 years for CRT with defibrillators (CRT-Ds) [3]. The clinical consequences of CIED infections can be critical, leading to considerable morbidity and mortality. An estimated 10%-30% of patients who develop infections following CIED implantation may experience fatal outcomes, especially those with systemic infections [4]. These findings underscore the need for early detection, robust preventive strategies, and timely, effective management of post-implantation infections. Given the increasing use of CIEDs and the associated rise in infection-related complications, continued research and clinical vigilance are critical to lowering infection rates, advancing device technology, and improving patient outcomes [1]. Potential strategies include refinement of implantation techniques, adoption of antimicrobial-enhanced devices, and strict postoperative follow-up protocols.

## Case presentation

A 44-year-old man presented to the hospital on February 2, 2025, with complaints of fever, shortness of breath, fatigue, generalized weakness, and purulent discharge from a pacemaker incision site. According to the patient, these symptoms had been present for the past three days and had progressively worsened, prompting him to seek medical attention. His medical history was notable for dilated cardiomyopathy, arterial hypertension, mitral and tricuspid valve insufficiency, heart failure classified as NYHA Class III with reduced ejection fraction (HFrEF), and a prior myocardial infarction (MI). Due to a high risk of sudden cardiac death, a CRT-D (three-chamber) was implanted on August 25, 2023. On July 17, 2024, the patient was evaluated by an outpatient arrhythmologist for discharge at the device incision site. Wound revision was performed, and a sample of the exudate was sent for microbiological analysis, which revealed methicillin-resistant *Staphylococcus epidermidis *(MRSE). However, the patient was lost to follow-up and did not receive targeted antimicrobial therapy for the identified pathogen.

Upon presentation, the patient was febrile (38.2°C), tachypneic with a respiratory rate of 26 breaths per minute, and had an oxygen saturation of 88% on room air. Physical examination was significant for an open postoperative wound in the upper left chest with purulent discharge. Auscultation showed diminished breath sounds bilaterally. Hemodynamically, the patient was stable with a blood pressure of 100/74 mmHg and a heart rate of 117 beats per minute (bpm).

Initial laboratory investigations (Table 1) demonstrated leukocytosis (WBC 19.66 × 10⁹/L), markedly elevated C-reactive protein (CRP, 266.04 mg/L) and procalcitonin (PCT, 12.87 ng/mL), hypoxemia (pO₂, 68 mmHg), and elevated lactate levels (4.2 mmol/ L).

**Table 1:** Laboratory results during hospitalization WBC: white blood count, CRP: C-reactive protein, PCT: procalcitonin.

Test	Unit	Normal range	Day 1	Day 14	Day 32
WBC	10^9^/L	4.00-9.00	19.66	8.62	6.08
Neutrophils	10^9^/L	2.00-6.80	18.8	6.38	4.13
CRP	mg/L	<5	266.04	126.36	56.78
PCT	ng/mL	<0.5	12.87	0.469	0.189
Lactate	mmol/L	0.9-1.7	4.2	1.7	0.7
pO₂	mmHg	83-108	68	71	102

Initial echocardiography findings

**Figure 1:**
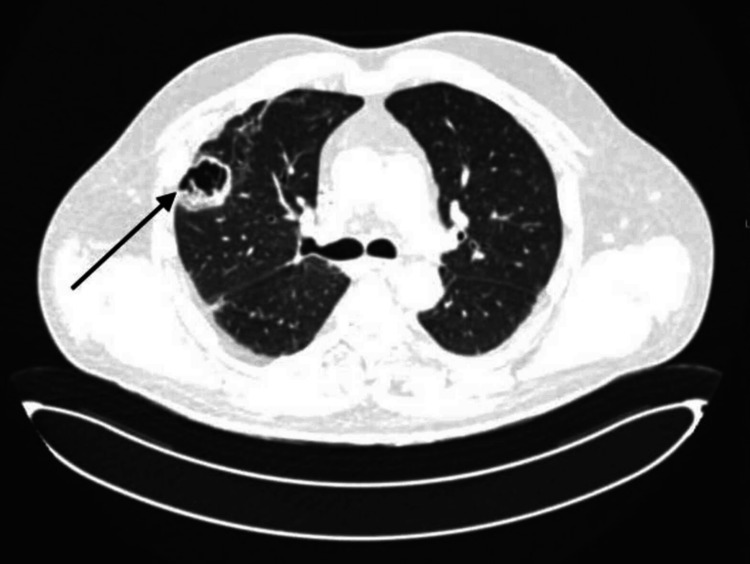
Thick-walled air cavities (black arrow)

**Figure 2:**
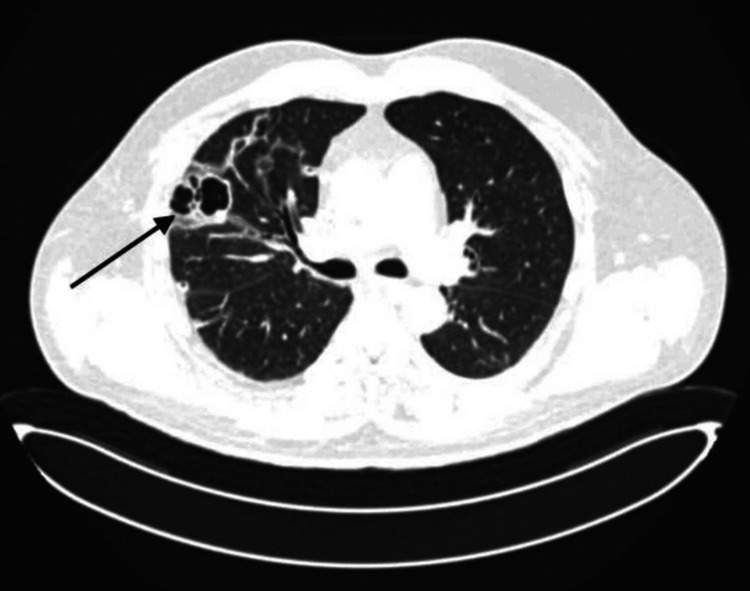
Multicystic septated cavity (black arrow)

**Figure 3:**
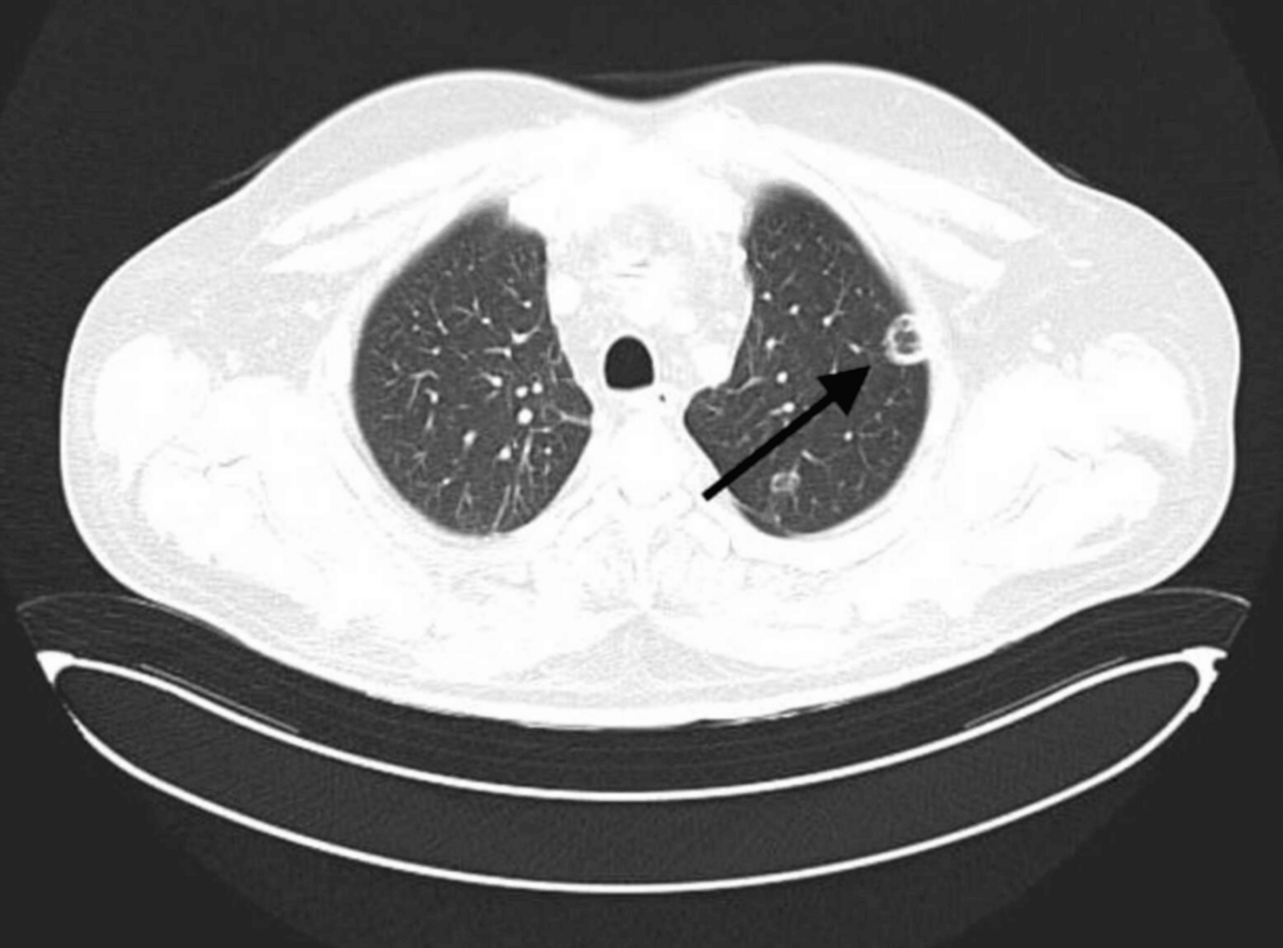
Small nodule (black arrow)

Echocardiography revealed a dilated left ventricle with moderately reduced systolic function and an ejection fraction of 44%. Global contractility showed a diffuse decrease. The left atrium was mildly dilated, with evidence of diastolic dysfunction and impaired relaxation. The valvular apparatus showed moderate mitral regurgitation (+2/+4) and mild tricuspid and aortic regurgitation (+1/+4). The right ventricle was mildly dilated, while the right atrial dimensions remained within normal limits. The systolic function of the right heart appeared mildly decreased. Pericardial effusion was detected around the right heart chambers, with a maximum separation of 1 cm. Based on clinical presentation, laboratory results, and application of the rapid Sequential Organ Failure Assessment (SOFA) criteria, a diagnosis of sepsis was established. Blood, wound exudate, and urine samples were sent for culture and sensitivity testing. Empirical broad-spectrum antimicrobial therapy was initiated with cefepime (2 g every 8 hours) and vancomycin (1 g every 12 hours). To evaluate the extent of infection and rule out pulmonary embolism (PE), chest computed tomography (CT) with pulmonary angiography was performed. No thoracic deformities or soft tissue abnormalities were noted. Cardiomegaly and pericardial effusion (9 mm separation) were present. In the dorsal region of the left apical lung, several thick-walled air-filled cavities were observed, the largest measuring 2.5 cm with thin septations and wall thickness of 3 mm (Figure 1). Additional smaller cavities were identified in the lingular segment and lower lobe. On the right, similar cavitary lesions were found, including a large multicystic, septated cavity in the upper lobe interlobar pleura, measuring 3.9-4.2 cm in diameter with wall thickness up to 7 mm (Figure 2). While no acute infiltrative changes were seen around the cavities, dull, coarse fibrous adhesions were noted. Moreover, several small nodular opacities (≤5 mm) were observed near the interlobar fissures in the upper and lower lobes of the right lung (Figure 3).

**Figure 4:**
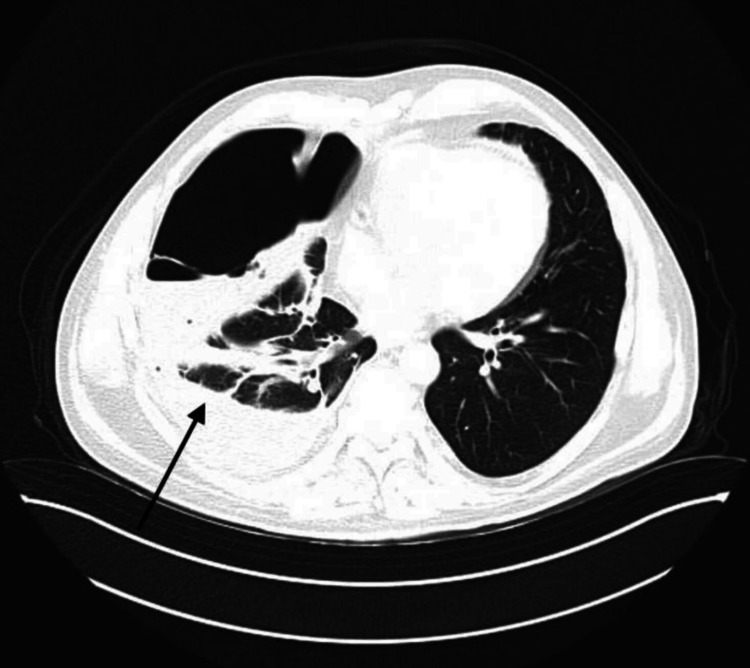
Area with dense fluid and air content (black arrow)

Given the complexity of the case, a multidisciplinary team meeting involving cardiologists, cardiothoracic surgeons, and infectious disease specialists was convened. Diagnostic samples were sent to rule out tuberculosis, based on the cavitary changes observed in the lungs. CRT-D extraction was scheduled. On February 4, 2025, the patient underwent CRT-D removal with lead extraction under general anesthesia. The surgical site was cleaned with Betadine three times after the dressing was removed. Sutures were taken out, and a breach in skin integrity was observed medially at the incision site. A clot-like mass was evacuated, and the wound was treated with Betadine and subsequently closed, with wound edges approximated and an aseptic dressing applied. Bacteriological testing returned negative for tuberculosis; however, blood cultures and incision site cultures were both positive for methicillin-resistant *Staphylococcus aureus* (MRSA). These findings supported a diagnosis of CRT-D-associated infective endocarditis complicated by septic PE. As the previously administered antibiotics were active against MRSA, the therapeutic regimen was continued as initially prescribed. Despite treatment, the patient remained febrile, prompting escalation of antimicrobial therapy. Meropenem 2 g every 8 hours (extended infusion) and vancomycin 1 g every 12 hours were initiated. Clinical improvement followed: inflammatory markers declined, although the patient remained subfebrile. Vancomycin trough levels were found to be subtherapeutic, necessitating a dose adjustment to 1 g every 8 hours based on body weight. Following this adjustment, the patient’s condition significantly improved. Repeat echocardiography on February 16, 2025, showed no abnormalities in the pericardial or pleural cavities. After three weeks of intravenous antibiotic therapy, the patient was discharged on oral antimicrobials: trimethoprim-sulfamethoxazole, rifampicin, and fluconazole (the latter added due to detection of *Candida albicans* in urine). A follow-up chest CT scan was planned, and the patient was advised to consult a pulmonologist. An outpatient follow-up was scheduled for one week later. On February 25, during the outpatient visit, the patient reported new-onset fever (up to 38°C) and chills over the past two days. Surgical wound revision was performed, and suture removal was attempted. The site was draining mainly serous fluid. The wound was treated accordingly. The patient also reported dyspnea. A chest X-ray revealed hydropneumothorax, likely due to a rupture of a pulmonary bulla. Imaging showed two horizontal fluid levels in the right mid-lung field; the right lower lobe and costophrenic sinus appeared homogeneously obscured. Air was seen in the right pleural cavity, corresponding to the upper field. Approximately two-thirds of the right lung was collapsed, with a mild mediastinal shift to the left. The left lung was fully expanded with a clear sinus. The patient was referred to the emergency department for further evaluation. A chest CT scan and thoracic surgical consultation were requested. Blood test results (Table 1) showed leukocytosis (WBC 13.04 × 10⁹/L) and elevated CRP (144.44 mg/L). Intravenous antimicrobial therapy with meropenem, vancomycin, and fluconazole was resumed. Bacteriological analysis of pleural effusion, blood, and wound cultures returned negative. Chest CT demonstrated persistent parenchymal changes in the left lung consistent with chronic septic embolism. The right pleural cavity showed septations with non-homogeneous dense fluid and air-fluid levels. Anterior to the large chest muscle on the left, near the clavicle, a fluid-and-air collection was noted, which appeared to connect to the skin surface (Figure 4).

Since the location of the effusion was well-defined, the thoracic surgeon proceeded with a systematic evacuation of the pleural fluid. The procedure was successfully performed, and a drainage catheter was strategically placed in the right pleural space to facilitate effective fluid removal. The patient was admitted to the surgical department for close monitoring and ongoing clinical evaluation. On February 28, 2025, a follow-up chest X-ray confirmed the drainage catheter positioned in the right pleural cavity, with its tip projected at the middle lung field. The lung was nearly fully expanded. Homogeneous shadowing was observed along the pleural wall, with several small horizontal fluid levels present. The costophrenic sinus was obscured, consistent with hydrothorax. A slight leftward shift of the mediastinum was also observed. Non-homogeneous shadowing was identified medially in the lower right lung field and in the upper field of the left lung. As the initial drainage proved insufficient, a second chest tube was inserted dorsally to enhance fluid evacuation. Two days after placement of the second drain, the patient's clinical status improved significantly: the fever resolved, dyspnea subsided, inflammatory markers normalized, and the CRT-D incision site showed proper healing. Follow-up CT imaging confirmed the resolution of the effusion, with only minimal residual fluid. The patient was discharged eight days after hospitalization with the following recommendations: follow-up visits on the 8th, 10th, and 20th days; strict outpatient follow-up under the supervision of a thoracic surgeon and cardiologist; and continued wound and drainage care under close surgical oversight.

## Discussion

This case highlights the complexity and high-risk nature of managing pacemaker-associated infective endocarditis, particularly when complicated by septic complications. The increasing incidence of CIED-related infective endocarditis (CIED-IE), driven by the growing number of device implantations and inadequate post-implantation follow-up, emphasizes the critical need for prompt identification and a collaborative, multidisciplinary approach to treatment [4,5]. Reported rates of CIED-IE range from 0.5% to 2.2% per year, with associated mortality rates fluctuating between 10% and 30% [6]. While established prognostic factors include advanced age, stroke, CNS abscess, cardiac valve replacement, pulmonary embolism, and acute renal failure, this case emphasizes the critical role of rigorous postoperative monitoring in preventing delayed diagnosis and related complications [7]. Advanced imaging techniques, particularly pulmonary CT angiography, played a key role in detecting thromboembolic complications in this patient. These modalities are increasingly recognized for their significant role in the early detection and management of such complications [8]. The patient’s clinical improvement following prolonged antimicrobial therapy aligns with existing literature, which supports extended treatment courses for complex CIED infections. Additionally, evidence suggests that patients who receive appropriate follow-up imaging tend to have more favorable prognoses, further underscoring the importance of post-discharge surveillance [9]. While this case illustrates successful management, it also highlights ongoing uncertainties regarding the long-term risk of reinfection. Further research is needed to establish standardized protocols and optimize outcomes for patients at elevated risk.

## Conclusions

This case underscores the critical importance of vigilant monitoring and comprehensive management in patients with CIED-associated infections. The complexity of these infections requires a multidisciplinary approach to achieve optimal outcomes. Early detection, timely intervention, and extended follow-up surveillance are essential to minimizing the risk of reinfection and improving long-term prognosis. Given the potential for severe complications, standardized guidelines for follow-up care must be established, particularly for high-risk patients. Ongoing research and sustained clinical vigilance are key to refining management strategies and enhancing patient outcomes in this challenging area of cardiovascular care.
